# Dispersion of radiocesium-contaminated bottom sediment caused by heavy rainfall in Joso City, Japan

**DOI:** 10.1371/journal.pone.0171788

**Published:** 2017-02-24

**Authors:** Kazumasa Inoue, Moeko Arai, Masahiro Fukushi

**Affiliations:** Department of Radiological Sciences, Graduate School of Human Health Sciences, Tokyo Metropolitan University, Tokyo, Japan; University of South Carolina, UNITED STATES

## Abstract

A large-scale heavy rainfall disaster occurred in Joso City, Japan, in September 2015, and one third of the city area (40 km^2^) was flooded by the Kinu River. Artificial radionuclides such as ^134^Cs and ^137^Cs were known to have accumulated in the river bottom sediment after their release in the 2011 Fukushima Dai-ichi Nuclear Power Plant accident. It was thought that these radionuclides might have been dispersed by the rainfall disaster. A car-borne survey of absorbed dose rate in air had been made by the authors in Joso City in August 2015. Then, the present study made a second car-borne survey in October 2015, to evaluate changes in the rate after the rainfall disaster. The absorbed dose rate in air and the standard deviation (range) measured in the flooded areas of Joso City after the disaster were 68 ± 9 nGy h^-1^ (39–98 nGy h^-1^), which was 10% higher than the rate before it. Additionally, higher dose rates (> 60 nGy h^-1^) were observed for the flooded areas after the disaster; furthermore, up to 886 Bq kg^-1^ of activity concentration from ^134^Cs and ^137^Cs was observed in these flooded areas, and this was 11 times higher than the activity concentration before the disaster. These results suggested the dispersion of artificial radionuclides accumulated in the bottom sediment of the Kinu River after the Fukushima Daiichi Nuclear Power Plant accident occurred by the heavy rainfall disaster.

## Introduction

**Fig 1:**
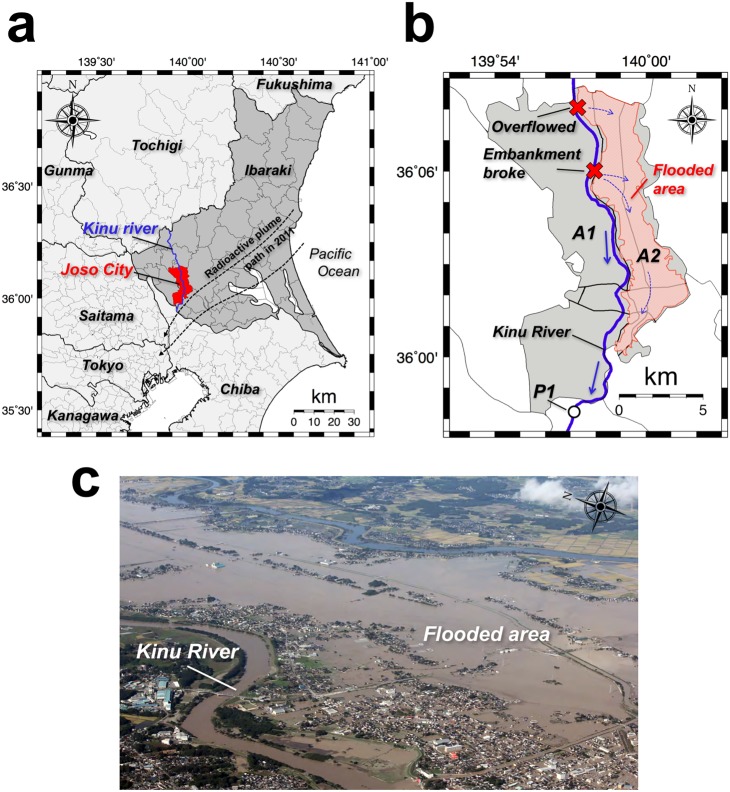
**(a) Map showing Joso City (red) and the Kinu River (blue) which flows through it. (b) The detailed map showing the part of the city that was flooded by the heavy rainfall disaster (pink) and the places were overflowing and breaking of the embankment occurred (x marks). (c) An aerial photograph of the disaster site [2].** These maps were drawn using the Generic Mapping Tools (GMT) created by Wessel and Smith [3].

Typhoon Etau caused extensive and destructive floods across eastern Japan on September 10 and 11, 2015. According to the latest press report from the Cabinet Office, Government of Japan [1], 19,723 houses and other structures were destroyed and 4,275 residents were rescued by authorities (summarized data as of February 19, 2016). Increased amounts of river water due to the continuous rainfall led to overflowing or breaking of embankments and caused major flooding in wide areas. Joso City, located northeast of Tokyo (Fig 1a), was the worst damaged area, and one third of the area (40 km^2^) was flooded (Fig 1b and 1c [2]). It took almost 10 days for flood water to recede by natural flow or by pumping.

It is known that large amounts of artificial radionuclides such as ^134^Cs and ^137^Cs which were released in the March 2011 accident at the Fukushima Daiichi Nuclear Power Plant (F1-NPP) were deposited on the ground of eastern Japan [4]. At the Joso City office on June 30, 2011, a dose rate at 1 m above the ground surface of 155 nGy h^-1^ was observed [5]. This dose rate is 3.1 times higher than the average dose rate in Japan (50 nGy h^-1^, uncertainty; 12.3%) before the F1-NPP accident [6]. Although 4.5 years have now passed since the F1-NPP accident, it is considered that the land-deposited artificial radionuclides have become accumulated in the bottom sediment of rivers, through the influence of weather conditions such as rainfall, and especially, because of the many mountainous regions, from which the land-deposited artificial radionuclides have easily flowed into the rivers [7].

In this paper, a car-borne survey was carried out to measure absorbed dose rate in air for all of Joso City after the heavy rainfall disaster, and a comparison was made with the value measured a month before it. Additionally, activity concentration in soil samples collected after the rainfall disaster was measured to verify the possible dispersion of artificial radionuclides which had accumulated in the bottom sediment of the Kinu River.

## Materials and methods

### Survey area

**Fig 2:**
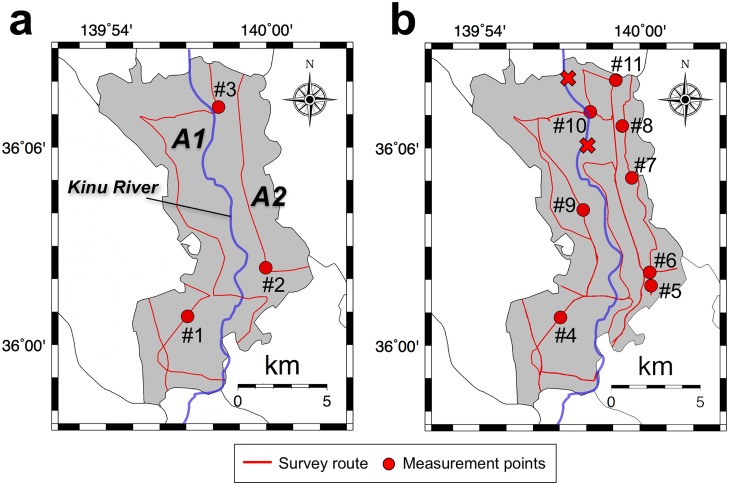
The survey routes for measuring the absorbed dose rates in Joso City before (a) and after (b) the heavy rainfall disaster. Car-borne surveys were carried out using a 3-in × 3-in NaI(Tl) scintillation spectrometer in August and October 2015. Total distances traveled were 59.3 km and 88.0 km, respectively. The fixed-point observations outside the car were also done for 10 min at 11 locations. These route maps were drawn using the Generic Mapping Tools (GMT) created by Wessel and Smith [3].

The absorbed dose rates in air (nGy h^-1^) from natural and artificial radionuclides (^40^K, ^238^U series, ^232^Th series, ^134^Cs and ^137^Cs) had been measured on August 11, 2015 (i.e., before the disaster) by the authors and they were measured for this study about possible radionuclide dispersion on October 28, 2015 (i.e., after the disaster) in Joso City, Ibaraki Prefecture (Fig 2a and 2b). These route maps were drawn using the Generic Mapping Tools (GMT) created by Wessel and Smith [3]. Main roads excluding expressways were used to the extent possible, primarily centered on residential areas. The measurement after the disaster was carried out after the flood water had receded and the land areas were dried out. The weather condition was sunny or cloudy throughout these measurement days. The survey routes before and after the disaster, respectively, were 59.3 km and 88.0 km long.

### Car-borne survey

It is advisable to carry out a wide range survey in a short measurement period at a certain level of the technique for reliable environmental radiation measurements. For this study, the car-borne survey after the disaster was carried out using a 3-in × 3-in NaI(Tl) scintillation spectrometer with a global positioning device (EMF-211, EMF Japan Co., Osaka, Japan). This survey system combined the NaI(Tl) scintillation spectrometer and a multichannel analyzer (GAMMA-RAD5, AMPTEK, Bedford, MA, USA). This was the same system as had been used by the authors in August and all procedures were the same in both surveys [8]. In brief, latitude and longitude at each measurement point were measured simultaneously with the recording of count rates in gamma-ray energies of 50 keV– 3.2 MeV. Measurement of the counts inside the car was performed every 30 s along the route and the car windows were kept closed during those measurements. Car speed was kept around 35 km h^-1^. The photon peaks of ^40^K (E_*γ*_ = 1.464 MeV) and ^208^Tl (E_*γ*_ = 2.615 MeV) were used for gamma-ray energy calibration from the channel number and gamma-ray energy before the measurements.

Since the NaI(Tl) scintillation spectrometer was placed inside the car, it is necessary to estimate a shielding factor in order to represent the count rate outside the car. The shielding factor was estimated after the heavy rainfall disaster by measurements at 8 locations as shown in Fig 2b (#4 –#11), both inside and outside the car. Measurements were recorded over consecutive 30 s intervals during a total recording period of 2 min. The shielding factor was calculated from the correlation between count rates inside and outside the car and the same value was used for data correction before and after the disaster since the same conditions were used.

The gamma-ray pulse height distributions were also measured in the survey after the disaster at the same 8 locations (#4 –#11) outside the car for 10 min to obtain a dose conversion factor (nGy h^-1^/cps). The NaI(Tl) scintillation spectrometer was positioned 1 m above the ground surface. The measured gamma-ray pulse height distributions were then unfolded using a 22 × 22 response matrix method [9] and absorbed dose rates in air were calculated because it is difficult to obtain the photon peaks in the 30-s measurement of the car-borne survey. Using these calculated absorbed dose rates in air, the dose conversion factor was then estimated from the correlation between dose rates and count rates. Both the obtained shielding factor and dose conversion factor were multiplied by count rates inside the car, and absorbed dose rates in air for the data measured before and after the disaster were calculated. The above fixed-point observations were carried out with the permission of permission of the land owners and it was also confirmed that the field studies did not involve endangered or protected species.

All obtained data from the two car-borne surveys were plotted as a distribution of absorbed dose rates in air in Joso City using GMT [3]. For a more detailed analysis, the absorbed dose rates in air from natural radionuclides (^40^K, ^238^U series and ^232^Th series) and artificial radionuclides (^134^Cs and ^137^Cs) were separated from gamma-ray pulse height distributions measured at 11 locations (#1 –#11 in Fig 2a and 2b) before and after the disaster using a 22 × 22 response matrix method. The detailed method of the analysis using a 22 × 22 response matrix method has been described previously [9].

### Activity measurement in soils

Soil samples were collected from a layer from the ground surface (i.e., 0 cm) to 10 cm depth at 8 locations (#4 –#11 in Fig 2b). The collected wet samples were naturally dried for a month. The dried samples were next sieved to get less than 2 mm sized particles which were then packed in a U-8 container (55 mm*ϕ* ×68 mm). Activity concentration measurements for ^134^Cs (E_*γ*_ = 605 and 796 keV) and ^137^Cs (E_*γ*_ = 662 keV) were carried out for 30,000 s per sample (sample weight: 90–103 g) using a high-purity germanium semiconductor detector (GMX10P, ORTEC, Oak Ridge, TN, USA) with a multichannel analyzer (MCA-7, SEIKO EG & G, Tokyo, Japan). The photon peak of ^137^Cs was used for gamma-ray energy calibration from the channel number and gamma-ray energy before the measurements.

## Results and discussion

### Shielding and dose conversion factors

**Fig 3:**
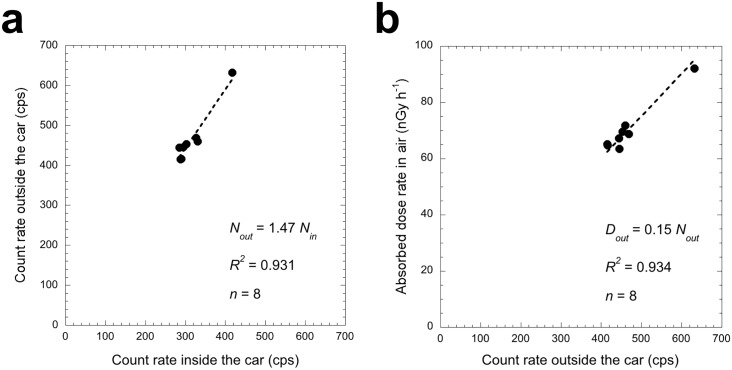
**Correlation between count rates inside and outside the car (*p* = 0.781) (a). Correlation between dose rates in air and count rates outside the car (*p* = 0.215) (b).** The absorbed dose rates in air were calculated using software that implemented the 22 × 22 response matrix method [9]. The slopes of these regression lines were used as the shielding factor and the dose conversion factor, respectively.

Fig 3a shows the correlation between count rates inside and outside the car obtained at 8 locations (#4 –#11 in Fig 2b). The shielding factor was calculated to be 1.47. The shielding factor is influenced by the type of car used in the survey, the number of passengers and the scintillation spectrometer position inside the car. In the present study, the same shielding factor was used for the data measured before and after the disaster because the measurements were carried out under the same conditions. Previous reports have given shielding factors ranging from 1.31 to 1.60 [8, 10–14], and the present value was within that range.

Fig 3b shows the correlation between dose rate (nGy h^-1^) calculated from the software using the 22 × 22 response matrix method [9] and total count rate outside the car. The dose rate conversion factor (nGy h^-1^/cps) was evaluated as 0.15. Since this dose conversion factor is an eigenvalue of each scintillation spectrometer, the same factor was used for the data measured before and after the disaster.

Both the calculated shielding and dose rate conversion factors were used with Eq (1) to calculate the absorbed dose rate in air (nGy h^-1^) outside the car 1 m above the ground surface (*D*_*out*_):

$$D_{out}=C_{in}\times 1.47\times 0.15$$ (1)

where *C*_*in*_ is count rate (cps) inside the car obtained by the car-borne for every 30 s.

### Changes of distribution of absorbed dose rates in air in Joso City

The average absorbed dose rates and standard deviations (ranges) measured before the heavy rainfall disaster were as follows: in the whole area of Joso City (*n* = 169), 60 ± 9 nGy h^-1^ (38–92 nGy h^-1^); A1 area (*n* = 108), 59 ± 8 nGy h^-1^ (38–83 nGy h^-1^); and A2 area (*n* = 61), 62 ± 9 nGy h^-1^ (48–92 nGy h^-1^). Before the disaster (i.e., 4.5 y after the F1-NPP accident), the dose rate measured in A2 area was slightly higher than that of A1 area. Furukawa and Shingaki [15] reported the absorbed dose rate in air in Joso City before the F1-NPP accident ranged from 30–40 nGy h^-1^. The present absorbed dose rate in air of 60 ± 9 nGy h^-1^ was 1.5–2.0 times larger compared to the literature value before the F1-NPP accident. Additionally, the average absorbed dose rates and standard deviations (ranges) measured after the disaster were as follows: in the whole area (*n* = 523), 66 ± 9 nGy h^-1^ (39–98 nGy h^-1^); A1 area (*n* = 221), 64 ± 8 nGy h^-1^ (39–83 nGy h^-1^); and A2 area (*n* = 302), 68 ± 9 nGy h^-1^ (39–98 nGy h^-1^). The absorbed dose rates in air were 10%, 9% and 10% higher compared to rates before the disaster, respectively, in the three areas. However, the percentage of increase in the absorbed dose rate in air was not significantly different between A1 and A2 areas.

**Fig 4:**
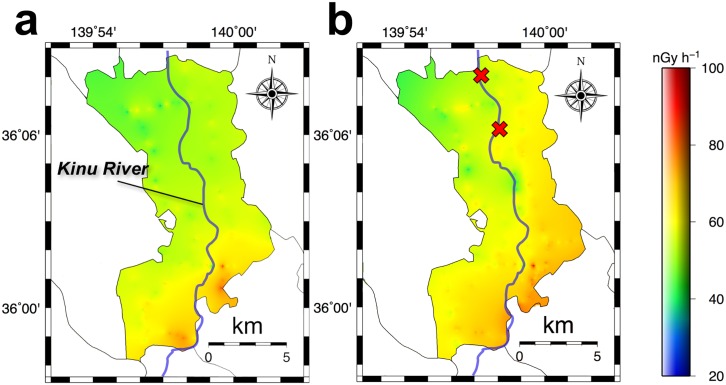
The distribution maps of absorbed dose rate in air in Joso City before (a) and after (b) the heavy rainfall disaster. The map for August 2015 (a) was drawn using 169 data and that for October 2015 (b) was drawn using 623 data.

Fig 4a shows the distribution map of absorbed dose rates in air in Joso City before the rainfall disaster. Higher absorbed dose rates in air were observed to be concentrated in the southern area of Joso City. According to air-borne radiation monitoring data obtained during August 2011 to May 2012 [16, 17], higher dose areas were observed from the northeastern area to the southwestern area of Ibaraki Prefecture (Fig 1a) and then they extended toward the southeast direction (i.e., eastern Tokyo and Saitama Prefecture) [18]. Thus, this shift might have influenced the absorbed dose rates of the southern area of Joso City. After the heavy rainfall disaster, higher absorbed dose rates were observed for all of A2 area and a part of A1 area (Fig 4b). Especially, a bigger change in the absorbed dose rate in air was observed for the flooded area (i.e., A2 area).

To allow a more detailed discussion, absorbed dose rates in air obtained from all the radionuclides, the natural radionuclides (^40^K, ^238^U series and ^232^Th series) and the artificial radionuclides (^134^Cs and ^137^Cs) at 11 locations are shown in Table 1. These dose rates were calculated using the 22 × 22 response matrix method [9]. The measurements of gamma-ray pulse height distributions at #1 and #4 in Fig 2a and 2b were for the same location. Other measurements, however, were carried out at different locations within < 100 m before and after the disaster because it was impossible to measure them at the exact same locations. The average dose rates and standard deviations from natural radionuclides before and after the disaster were 66 ± 6 and 61 ± 4 nGy h^-1^, respectively, so the dose rate decreased 8% after the disaster. According to the Joso City Office [19], many houses were washed away by the flooding (completely destroyed, 53 houses; large-scale destruction over half-collapsed, 1,581 houses; half-collapsed, 3,491 houses), and one third of the city area (40 km^2^) was flooded. Many natural radionuclides are contained in building materials and the ground. Therefore, the measured absorbed dose rates at 1 m above the surface of the ground depend on the amount of building materials and the percentage of water content in the ground which has a shielding effect. The measured dose rate after the disaster was slightly decreased compared to that before the disaster. On the other hand, average absorbed dose rate in air and standard deviations (range) from artificial radionuclides were increased from 1 ± 1 nGy h^-1^ (1–3 nGy h^-1^) to 9 ± 12 nGy h^-1^ (2–39 nGy h^-1^), and this resulted in a dose rate that was significantly increased after the heavy rainfall disaster.

**Table 1:** Absorbed dose rate in air from natural and artificial radionuclides before and after the 2015 heavy rainfall disaster.

No.^a^	Area^b^	Absorbed dose rate in air (nGy h^-1^)		
		All	Natural radionuclides	Artificial radionuclides
1	A1	73	73	0
2	A2	64	63	1
3	A2	66	63	3
4	A1	67	65	2
5	A2	93	53	39
6	A2	72	62	9
7	A2	70	65	4
8	A2	69	62	6
9	A1	65	62	3
10	Kinu River	65	64	2
11	A2	63	58	6

^a^ “No.” refers to the specific sampling point designations in Fig 2a and 2b.

^b^ “Area” refers to the measurement area designations in Fig 1b.

### Activity measurement in soils

The activity concentrations of ^134^Cs and ^137^Cs contained in soil samples that were collected at 8 locations (Fig 2b) after the rainfall disaster are shown in Table 2. The average activity concentration and standard deviation (range) for radiocesium (^134^Cs + ^137^Cs) were 247 ± 271 Bq kg^-1^ (63–886 Bq kg^-1^). According to the press report from the Ministry of the Environment [20], in August 2015, 103 Bq kg^-1^ of radiocesium (^134^Cs and ^137^Cs) was observed in bottom sediment collected at P1 (in Fig 1b) which was 10 km downstream from the center of Joso City. In addition, for October 28, 2015, the activity concentration in Joso City (at an unidentified location) could be estimated based on the results of a survey performed in August—September 2011 by the Ibaraki Prefecture Government [21]; this value was 81 Bq kg^-1^, considering activity decay.

**Table 2:** Activity concentrations of ^134^Cs and ^137^Cs in soil samples after the heavy rainfall disaster.

No.^a^	Area^b^	Activity concentrations (Bq kg^-1^)	
		^134^Cs	^137^Cs
4	A1	23	96
5	A2	39	165
6	A2	164	722
7	A2	45	192
8	A2	12	51
9	A1	18	81
10	Kinu River	55	232
11	A2	17	64

^a^ “No.” refers to the specific sampling point designations in Fig 2a and 2b.

^b^ “Area” refer to the measurement area designations in Fig 1b.

The activity concentrations measured at #5, #6 and #7 which were south of the place where the embankment broke were higher than before the disaster as described above [20, 21]. In addition, the activity concentrations for ^134^Cs and ^137^Cs at #6 were higher than their activity concentrations (55 and 287 Bq kg^-1^ in Table 2) found in the river bottom sediment collected at #10 (Fig 2b). Thus, it seemed that the river mud containing artificial radionuclides was dispersed to A2 area of Joso City by the flooding and the absorbed dose rate in air was increased. The data clearly indicated that the radiocesium (^134^Cs and ^137^Cs) deposited by the F1-NPP accident was easily transported by a natural disaster, and the activity concentrations of artificial radionuclides were significantly increased at the place where they were transported to.

### Uncertainty on measurements of absorbed dose rates in air

The relative standard uncertainties for the shielding factor, dose conversion factor, traceability of the dose rate, and the dose calculation procedure by the response matrix method were given as 11.0%, 9.3%, 4.1% (*k* = 2) (Pony Industry Co., Ltd., Osaka, Japan), and 5.0% (EMF Japan Co., Osaka, Japan), respectively. Additionally, the range of relative standard uncertainty for 30 s measurements was also given as 1.7%– 2.7%. Thus, the maximum combined relative standard uncertainty to the estimated ambient dose rate in this car-borne survey was calculated to be 15.8%. For measurements in metropolitan Tokyo using the same dosimeter and same measurement method [18], the obtained relative standard uncertainties for the shielding factor and dose conversion factor were 7.2% and 5.5%, respectively. Those uncertainties were lower compared to the present study. This might be due to the difference in the number of measurement locations (*n* = 62 vs. *n* = 8). However, the dose conversion factor obtained in the present study was the same 0.15 nGy h^-1^/cps as previously obtained [18]. In addition, the obtained shielding factor was an appropriate value as described earlier. Thus, the use of both factors seemed to be appropriate for the present study.

The obtained maximum combined relative standard uncertainty in the measurement for Joso City using car-borne survey technique (15.8%) was higher than the changed percentage of absorbed dose rate in air from natural and artificial radionuclides before and after the heavy rainfall disaster (10%). Here, the absorbed dose rate in air measured for Arakawa Ward, Tokyo (S1 Fig) on a monthly basis in 2015 using the same dosimeter and the same measurement method is shown in S2 Fig (*n* = 256). The same dose rates were observed throughout the year; there was no seasonal variation. Although the maximum combined relative standard uncertainty was 15.9% in the Arakawa Ward measurement, the same dose rates were obtained for the absorbed dose rate in air from the natural and artificial radionuclides. For a more detailed analysis, the absorbed dose rates in air from natural and artificial radionuclides measured by fixed-point observations (*n* = 13) were separated by the response matrix method, because the dose rates measured for every 30 s by the car-borne survey included the effect of the artificial radionuclides, and those calculated values are shown in S1 Table. The same average absorbed dose rates in air and standard deviations from natural radionuclides were observed throughout the year. On the other hand, the average absorbed dose rates in air from artificial radionuclides measured before (*n* = 3) the rainfall disaster (1 ± 1 nGy h^-1^) in this study were significantly increased after (*n* = 8) the disaster (9 ± 12 nGy h^-1^). The combined relative standard uncertainty on these fixed-point observations was calculated to be 6.5% because the shielding and dose conversion factors are not required, and dose rate was measured for 10 min at a fixed point resulting in a percentage of increased dose rate well above the uncertainty value. The average absorbed dose rate in air from natural radionuclides was conversely decreased from 66 ± 6 to 61 ± 4 nGy h^-1^; as a consequence, dose rate decreased 8% after the disaster. While the car-borne survey technique was useful to observe dose rate distribution for a large area, it could not detect changes of both dose rates from natural and artificial radionuclides. Thus, the lower change in ambient dose rate in air was observed compared to the combined relative standard uncertainty of the car-borne survey technique. Based on these results obtained from the same dosimeter and the same measurement method, it was suggested that the dispersion of artificial radionuclides accumulated in the bottom sediment of the Kinu River after the F1-NPP accident occurred by the heavy rainfall disaster.

## Conclusion

The absorbed dose rates in air before and after a heavy rainfall disaster were measured using the car-borne survey technique with a NaI(Tl) scintillation spectrometer and changes in the values were observed. The average dose rate in the flooded area of Joso City was increased from 60 ± 9 nGy h^-1^ to 68 ± 9 nGy h^-1^, and that led to the 10% increase of dose rate compared to the rate before the disaster. However, the change in absorbed dose rate in air before and after the disaster was less than the combined relative standard uncertainty (15.9%). The absorbed dose rate in air from natural radionuclides measured by fixed-point observations was decreased and that of artificial radionuclides was locally increased after the heavy rainfall disaster. While the car-borne survey technique was useful to estimate the change of dose distribution before and after the disaster for a large area, it was an unsuitable technique to observe the local change in absorbed dose rate in air. On the other hand, the average absorbed dose rate from artificial radionuclides measured by fixed-point observations was significantly increased from 1 ± 1 nGy h^-1^ to 9 ± 12 nGy h^-1^, and this resulted in a change that was larger than the combined relative standard uncertainty (6.5%). Additionally, higher activity concentrations from artificial radionuclides were observed in soil samples collected south of the place where the Kinu River embankment broke and extensive flooding occurred. These activity concentrations were a maximum of 11 times higher compared to the values before the disaster. Thus, the dispersion of artificial radionuclides accumulated in the bottom sediment of the Kinu River was suggested after the heavy rainfall disaster in Joso City. Based on these results, the data showed increased radiation doses to members of the general public due to the resuspension of radioactively-contaminated river sediments.

## Supporting information

S1 Fig The location of Arakawa Ward in Tokyo (a) and survey route for measuring the absorbed dose rate in air (b). (TIF)

S2 Fig The seasonal variation of absorbed dose rate in air in Arakawa Ward, Japan in 2015. (TIF)

S1 Table Absorbed dose rate in air from natural and artificial radionuclides measured by fixed-point observations in Arakawa Ward, Japan in 2015. (DOCX)
