# Supplementary figures and images for: Dispersion of radiocesium-contaminated bottom sediment caused by heavy rainfall in Joso City, Japan
Source: PLoS One. 2017 Feb 24;12(2):e0171788. doi: 10.1371/journal.pone.0171788 (PMC5325223; doi:10.1371/journal.pone.0171788)

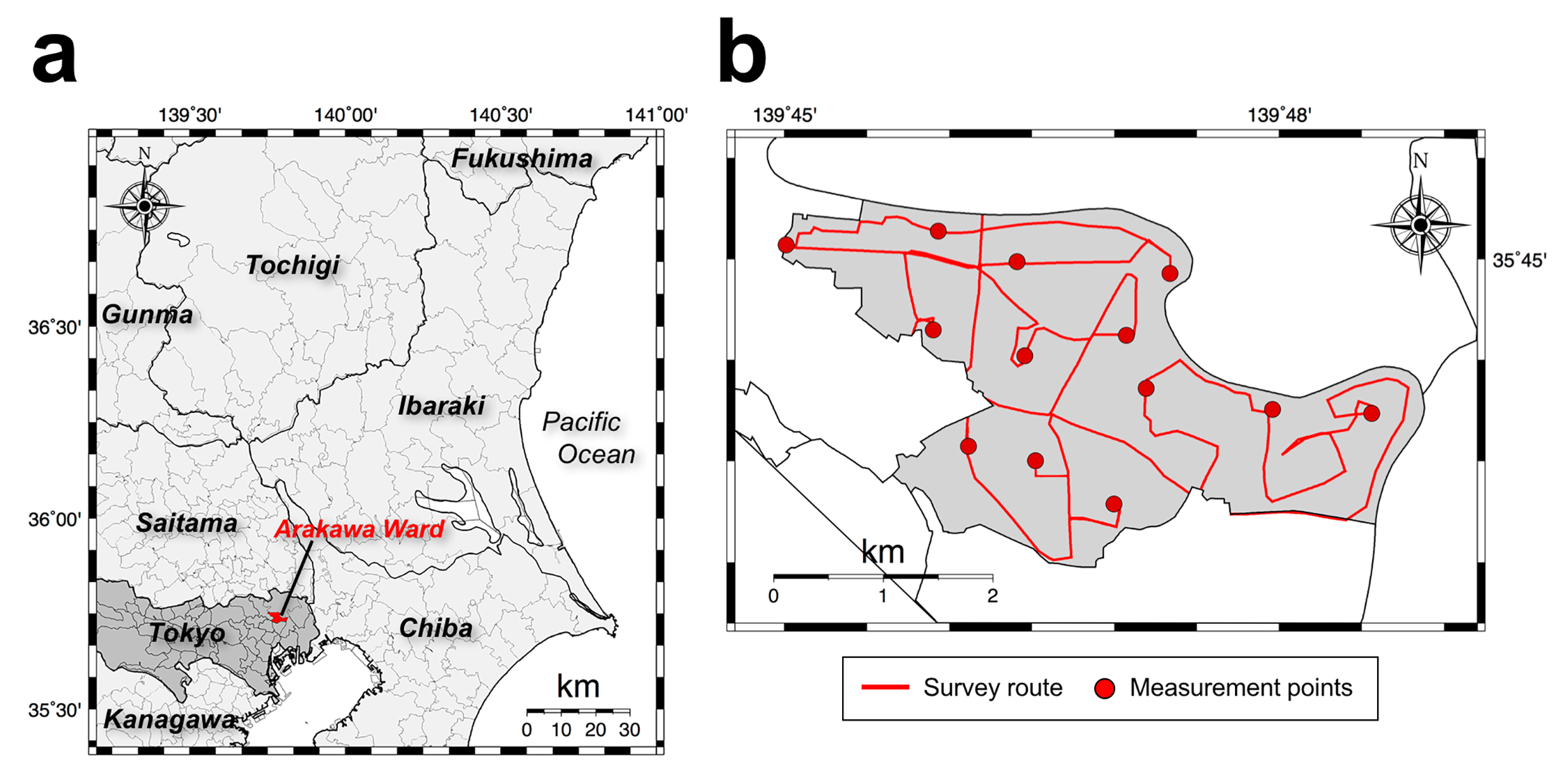

Supplement: S1 Fig — (TIF) [file pone.0171788.s001.tif]

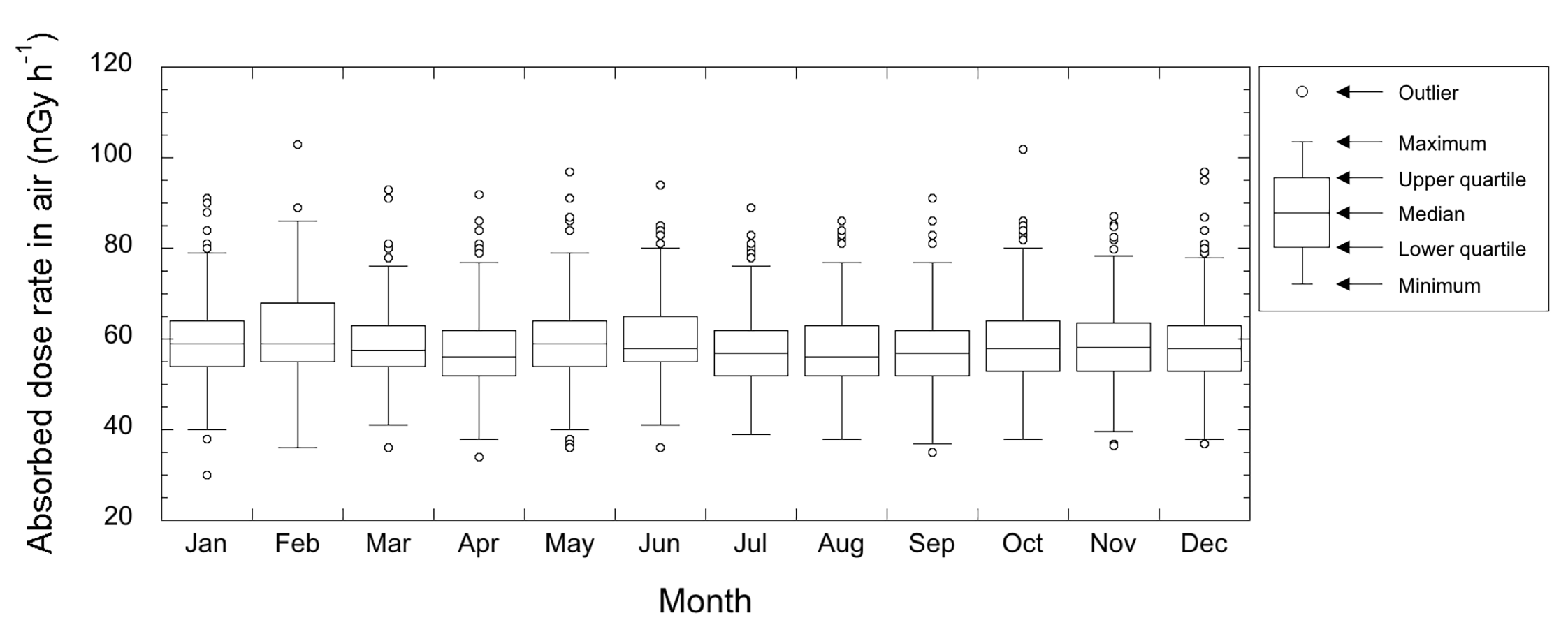

Supplement: S2 Fig — (TIF) [file pone.0171788.s002.tif]
