# Supplementary material for: Dispersion of radiocesium-contaminated bottom sediment caused by heavy rainfall in Joso City, Japan
Source: PLoS One. 2017 Feb 24;12(2):e0171788. doi: 10.1371/journal.pone.0171788 (PMC5325223; doi:10.1371/journal.pone.0171788)
Supplement: S1 Table — (DOCX) [file pone.0171788.s003.docx]

| **Month** | **Absorbed dose rate in air (nGy h^-1^)** | | |
| --- | --- | --- | --- |
|  | **All** | **Natural radionuclides** | **Artificial radionuclides** |
| Jan. | 57±8 | 48±8 | 9±5 |
| Feb. | 56±7 | 48±7 | 8±5 |
| Mar. | 57±8 | 51±7 | 6±4 |
| Apr. | 55±6 | 48±6 | 7±7 |
| May | 56±7 | 50±7 | 6±5 |
| June | 56±7 | 50±7 | 6±4 |
| July | 56±7 | 50±7 | 6±5 |
| Aug. | 56±7 | 50±8 | 6±4 |
| Sept. | 56±7 | 50±6 | 6±5 |
| Oct. | 56±6 | 51±7 | 5±4 |
| Nov. | 55±6 | 50±7 | 5±4 |
| Dec. | 57±7 | 50±7 | 7±4 |
